# DMAS Beamforming with Complementary Subset Transmit for Ultrasound Coherence-Based Power Doppler Detection in Multi-Angle Plane-Wave Imaging

**DOI:** 10.3390/s21144856

**Published:** 2021-07-16

**Authors:** Che-Chou Shen, Yen-Chen Chu

**Affiliations:** Department of Electrical Engineering, National Taiwan University of Science and Technology, Taipei 106335, Taiwan; m10807306@mail.ntust.edu.tw

**Keywords:** delay-and-sum (DAS), delay-multiply-and-sum (DMAS), signal coherence, power doppler detection, plane-wave (PW) imaging, complementary subset transmit (CST), coherent plane-wave compounding (CPWC)

## Abstract

Conventional ultrasonic coherent plane-wave (PW) compounding corresponds to Delay-and-Sum (DAS) beamforming of low-resolution images from distinct PW transmit angles. Nonetheless, the trade-off between the level of clutter artifacts and the number of PW transmit angle may compromise the image quality in ultrafast acquisition. Delay-Multiply-and-Sum (DMAS) beamforming in the dimension of PW transmit angle is capable of suppressing clutter interference and is readily compatible with the conventional method. In DMAS, a tunable *p* value is used to modulate the signal coherence estimated from the low-resolution images to produce the final high-resolution output and does not require huge memory allocation to record all the received channel data in multi-angle PW imaging. In this study, DMAS beamforming is used to construct a novel coherence-based power Doppler detection together with the complementary subset transmit (CST) technique to further reduce the noise level. For *p* = 2.0 as an example, simulation results indicate that the DMAS beamforming alone can improve the Doppler SNR by 8.2 dB compared to DAS counterpart. Another 6-dB increase in Doppler SNR can be further obtained when the CST technique is combined with DMAS beamforming with sufficient ensemble averaging. The CST technique can also be performed with DAS beamforming, though the improvement in Doppler SNR and CNR is relatively minor. Experimental results also agree with the simulations. Nonetheless, since the DMAS beamforming involves multiplicative operation, clutter filtering in the ensemble direction has to be performed on the low-resolution images before DMAS to remove the stationary tissue without coupling from the flow signal.

## 1. Background

Delay-and-Sum (DAS) beamforming is routinely adopted to produce image output in medical ultrasound imaging by compensating the time delay of the received echo according to the geometric path of propagation before coherent summation [1]. However, it suffers from intrinsic limitations such as insufficient image resolution and noticeable off-axis clutter. For plane-wave (PW) imaging which depends on the unfocused transmit wave to illuminate a wide field-of-view [2], these limitations are notably evident. In single-angle PW imaging, the received backscattered echoes from one PW transmit event are processed using DAS beamforming in the direction of receiving channel to generate the corresponding low-resolution image at frame rate on the order of kHz. Therefore, PW imaging is also referred as ultrafast imaging. The image quality of PW imaging can be improved by coherent plane wave compounding (CPWC) in which synthetic transmit focusing is achieved from multi-angle PW transmit [3,4]. Specifically, low-resolution images are firstly acquired from several PW transmit angles and then coherently combined to achieve the final high-resolution CPWC image. Considering an imaging depth of 75 mm, the pulse-repetition-interval (PRI) for pulse-echo imaging will be 100 μs with the sound velocity of 1.5 mm/μs. This corresponds to a frame rate of 10 kHz for low-resolution PW imaging. Assuming that every 10 low-resolution images is coherently combined to improve the image quality, the resultant high-resolution CPWC images will be produced at a frame rate of 1 kHz. With this high frame rate, CPWC imaging has been utilized to detect the motion of imaged objects in transient elastography [4,5] and Doppler flow imaging [6,7,8,9,10,11,12,13]. However, it should be noted that the image quality in CPWC imaging relies on the number of low-resolution images involved in the compounding, and thus an inevitable trade-off between the image quality and the frame rate exists. In other words, with just a few PW transmit angles, the image quality in CPWC imaging is generally unsatisfactory due to the presence of high clutter artifacts.

Note that the aforementioned CPWC imaging for each image pixel is actually performed by coherently summing all the received channel data from all the PW transmit angles. These data can be represented as an echo matrix comprising two dimensions of the PW transmit angle and the receiving channel. Thus, the CPWC imaging is actually constructed using two-dimensional DAS beamforming. Note that the signal coherence of the two-dimensional echo matrix can be used to reject low-coherence clutters and thermal noises to further improve the multi-angle PW image quality. One particular example is Delay-Multiply-and-Sum (DMAS) beamforming. Originally, DMAS beamforming is developed to extract the signal coherence in the dimension of receiving channel by multiplying the received echoes between every possible channel pair after time compensation [14]. In order to improve the computational efficiency of the original DMAS beamforming, alternative high-order versions of DMAS beamforming have been recently proposed with flexibly tunable image quality in [15,16]. Take the BB-DMAS [16] as an example, where a rational *p* value is used to represent the order of DMAS beamforming. Note that a higher image quality can be achieved by adopting a higher *p* value to emphasize more spatial coherence in DMAS beamforming. The implementation of BB-DMAS beamforming involves the magnitude scaling of time-delayed channel signal by *p*-th root and the subsequent *p*-th power after channel sum. DMAS beamforming has also been extended to multi-angle PW imaging by extracting the signal coherence of two-dimensional echo matrix. In [17], DMAS beamforming is applied in the dimension of receiving channel to exploit the spatial coherence of synthesized echoes in different channels. The synthesized channel data is produced by summing the echo matrix in the dimension of PW transmit angle for synthetic transmit focusing as in CPWC imaging [17]. On the contrary, the two-dimensional spatial coherence can be also derived directly from the entire echo matrix using echoes in both dimensions [18].

In this study, a novel coherence-based DMAS power Doppler detection together with complementary subset transmit (CST) is proposed for multi-angle PW imaging. Power Doppler provides essential information of the backscattered power of flow signal and generally has higher sensitivity to small vessels than color Doppler [19,20]. This is because these small vessels may not be detectable using velocity estimation in color Doppler due to noises. The proposed method firstly adopts DMAS beamforming in the dimension of PW transmit angle to suppress the background noise and clutter. Then, the CST technique is used to further reduce the noise level in power Doppler detection by correlation of two complementary DMAS signals. Unlike the coherent flow power Doppler (CFPD) method [21,22] that relies on short-lag spatial coherence [23] to extract the coherence of blood flow signal in the dimension of receiving channel, the proposed DMAS beamforming is based on the signal coherence among low-resolution images from distinct PW transmit angles. It is compatible with current CPWC imaging and does not require huge memory allocation to retain the entire channel data in the echo matrix. In other words, the delayed channel data is firstly summed to one low-resolution image pixel. Then, DMAS beamforming in the dimension of PW transmit angle can be performed by magnitude-scaling these low-resolution images from distinct PW transmit angles before restoring the signal dimensionality to produce the high-resolution image after coherent compounding. Section 2 introduces the basics of DMAS beamforming in the dimension of PW transmit angle for power Doppler detection and the subsequent implementation of CST technique. Simulation methods in this study are described in detail in Section 3, together with experimental setups. In Section 4, image quality of the proposed DMAS-based power Doppler detection is quantitatively presented. Section 5 concludes our results with discussions.

## 2. Theory

### 2.1. Basics of Power Doppler Detection

In Doppler ultrasound imaging, the motion of red blood cells in the vessel is detected by repetitive pulse transmissions to observe the temporal variations of backscattered signals. For each image pixel, the recorded signal corresponding to the *f*-th pulse transmission is generally referred to as the *f*-th Doppler ensemble where *f* is the index of ensemble (*f* = 1, 2,…, *F*). In other words, there are a total of *F* ensembles available for velocity estimation in color Doppler and/or power estimation in power Doppler. Note that, in order to separate the blood flow signal from the stationary tissue signal and the thermal noises, a temporal clutter filter has to be applied to the Doppler ensembles to extract signal components with frequencies within a low-order threshold and a high-order threshold. In other words, the Doppler ensembles is band-pass filtered in the ensemble direction which is also called the slow-time direction. In this study, the band-pass clutter filtering is implemented using singular-value decomposition (SVD) [24,25] whose low-order and high-order thresholds are both adaptively determined.

Conventionally, the blood flow estimation can be achieved by autocorrelation of these Doppler ensembles as proposed in [26]. Specifically, the Doppler power is represented using the zero-lag autocorrelation as

(1) $${\mathrm{PD}}_{}=\sum_{f=1}^{F}{\left|y^{f}\right|}^{2}$$

where $y^{f}$ is the *f*-th Doppler ensemble after beamforming. Note that the Doppler power in Equation (1) is simply the summation of the squared magnitude of each Doppler ensemble.

In multi-angle PW imaging, the DAS beamforming (i.e., CPWC image) is the coherent summation of low-resolution image pixels from distinct PW transmit angles. Therefore, given the low-resolution image pixel as $x_{m}^{}$ where *m* is the index of PW transmit angle (*m* = 1, 2,…, *M*), the output of DAS beamforming is represented as

(2) $$y_{\mathrm{DAS}}=\sum_{m=1}^{M}x_{m}^{}$$

Note that the summation in Equation (2) is to produce the high-resolution CPWC image. After substituting Equation (2) into Equation (1), the conventional power Doppler detection of DAS beamforming in CPWC imaging is calculated as

(3) $${\mathrm{PD}}_{\mathrm{DAS}}=\sum_{f=1}^{F}\left({\left|\sum_{m=1}^{M}x_{m}^{}\right|}^{2}\right)$$

In other words, the power of the high-resolution image is summed among ensembles to provide the final power Doppler estimation of DAS beamforming.

### 2.2. Power Doppler Detection of DMAS Beamforming 

The DMAS beamforming in this study is implemented using baseband data to eliminate the need for oversampling of radio-frequency waveform [16]. Specifically, when the baseband data for low-resolution image pixel from the *m*-th PW transmit angle (i.e., $x_{m}^{}$ in Equation (2)) is represented as $x_{m}^{}=a_{m}e^{j\phi_{m}}$, DMAS beamforming in the dimension of PW transmit angle is performed by first maintaining the phase of low-resolution pixel but adopting the *p*-th root to scale the pixel magnitude as ${\overset{\wedge }{x}}_{m}=\sqrt[{p}]{a_{m}}e^{j\phi_{m}}$. Then, the *p*-th power of the summation of magnitude-scaled low-resolution image pixels from all the available PW transmit angles is performed to produce the final high-resolution image pixel. In other words, DMAS beamforming in the dimension of PW transmit angle can be defined as

(4) $$y_{\mathrm{DMAS}}={\left(\sum_{m=1}^{M}{\overset{\wedge }{x}}_{m}\right)}^{p}={\left(w^{H}\overset{\wedge }{x}\right)}^{p}$$

where

$$w={\left[\begin{array}{ccccc} 1 & 1 & 1 & \ldots & 1 \end{array}\right]}^{T}=1\;$$

$$\overset{\wedge }{x}={\left[\begin{array}{ccccc} {\overset{\wedge }{x}}_{1} & {\overset{\wedge }{x}}_{2} & {\overset{\wedge }{x}}_{3} & \ldots & {\overset{\wedge }{x}}_{M} \end{array}\right]}^{T}$$

Here, the symbol *H* represents Hermitian transpose and the real-valued weighting vector w is actually a unity vector 1 to equally emphasize the contribution from all the available PW transmit angles. For power Doppler detection, the magnitude of DMAS image is averaged among consecutive ensembles before the calculation of image power. In other words, the power Doppler of DMAS beamforming in this study is formulated as

(5) $${\mathrm{PD}}_{\mathrm{DMAS}}={\left(\sum_{f=1}^{F}\left|y_{\mathrm{DMAS}}\right|\right)}^{2}$$

Though the proposed DMAS beamforming is also applicable to B-mode imaging, it should be noted that the DMAS beamforming in this study is calculated from low-resolution images after SVD clutter filtering in order to remove both stationary tissue and noises for power Doppler estimation.

### 2.3. Power Doppler Detection of DMAS Beamforming with CST (DMAS-CST)

DMAS beamforming with CST technique depends on DMAS signals from two subsets of PW transmit angles. The idea of complementary subset is similar to that in [27,28,29] but is defined in the dimension of PW transmit angle instead of receiving channel. Specifically, DMAS beamforming is performed using the available PW transmit angles in each transmit subset and the beamforming output is denoted as $y_{\mathrm{DMAS}1}$ and $y_{\mathrm{DMAS}2}$, respectively, for subset 1 and subset 2:

$$\begin{array}{l} y_{\mathrm{DMAS}1}={\left(w_{1}^{H}\overset{\wedge }{x}\right)}^{p}\\ y_{\mathrm{DMAS}2}={\left(w_{2}^{H}\overset{\wedge }{x}\right)}^{p} \end{array}$$

where the weighting vector $w_{1}$ for subset 1 is related to the weighting vector $w_{2}$ for subset 2 by $w_{1}=1-w_{2}$ to ensure the complementary property. Note that, when the total number of available PW transmit angle is odd-valued, the subset 1 and 2 can share one specific PW transmit angle to equalize the number of PW transmit angle in each subset. For example, when there are totally seven PW angles in the transmit sequence, $w_{1}$ and $w_{2}$ can be respectively defined as [1 1 1 0.5 0 0 0] and [0 0 0 0.5 1 1 1] so that each subset comprises half of the total PW transmit angles. For even number of PW transmit angle, on the other hand, any PW transmit angle should belong to either one of the two complementary subsets. The two DMAS signals are then correlated to reduce the noise level and a square root of the correlation is performed to restore the dimensionality of DMAS signal. In other words, the DMAS-CST beamforming can be formulated as

(6) $$y_{\mathrm{DMAS}-\mathrm{CST}}=\sqrt{y_{\mathrm{DMAS}1}^{}\;y_{\mathrm{DMAS}2}^{*}}=\;{\left(w_{1}^{H}\;R\;w_{2}^{}\right)}^{\frac{p}{2}}$$

where the symbol * is for complex conjugate and $R=\overset{\wedge }{x}{\overset{\wedge }{x}}^{H}$ is the autocorrelation matrix of magnitude-scaled low-resolution image pixels from different PW transmit angles (i.e., $\overset{\wedge }{x}$ in Equation (4)). Note that the power Doppler detection of DMAS-CST beamforming is also implemented by ensemble averaging of $y_{\mathrm{DMAS}-\mathrm{CST}}$ before power estimation as

(7) $${\mathrm{PD}}_{\mathrm{DMAS}-\mathrm{CST}}={\left(\sum_{f=1}^{F}y_{\mathrm{DMAS}-\mathrm{CST}}\right)}^{}{\left(\sum_{f=1}^{F}y_{\mathrm{DMAS}-\mathrm{CST}}\right)}^{*}$$

**Figure 1:**
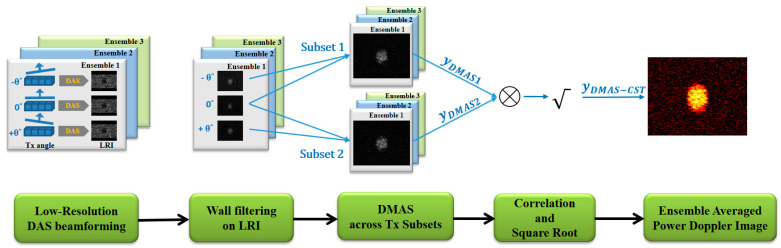
Schematic diagram of DMAS-CST beamforming for power Doppler detection in multi-angle PW imaging. Note that the wall filtering is performed on low-resolution images of each PW transmit angle before DMAS beamforming.

The signal flowchart of DMAS-CST beamforming for power Doppler estimation is schematically represented in Figure 1. It should be noted that, when both $w_{1}$ and $w_{2}$ are replaced with the unity vector 1, power Doppler of DMAS-CST beamforming in Equation (7) will degenerate to that of DMAS beamforming in Equation (5). In other words, the original DMAS beamforming can be understood as a special case of DMAS-CST beamforming. Moreover, it is expected that the achievable noise reduction in DMAS-CST beamforming should rely on the number of ensembles. Note that the complementary weighting vectors $w_{1}$ and $w_{2}$ can effectively eliminate the uncorrelated noise only when the noise component in the autocorrelation matrix **R** is diagonal. However, this statistically demands sufficient ensemble averaging for the noise component to converge to $\sigma_{N}^{2}I$ where **I** is the identity matrix and $\sigma_{N}^{2}$ is the noise variance of magnitude-scaled low-resolution image.

## 3. Methods

### 3.1. Simulation Setup

**Figure 2:**
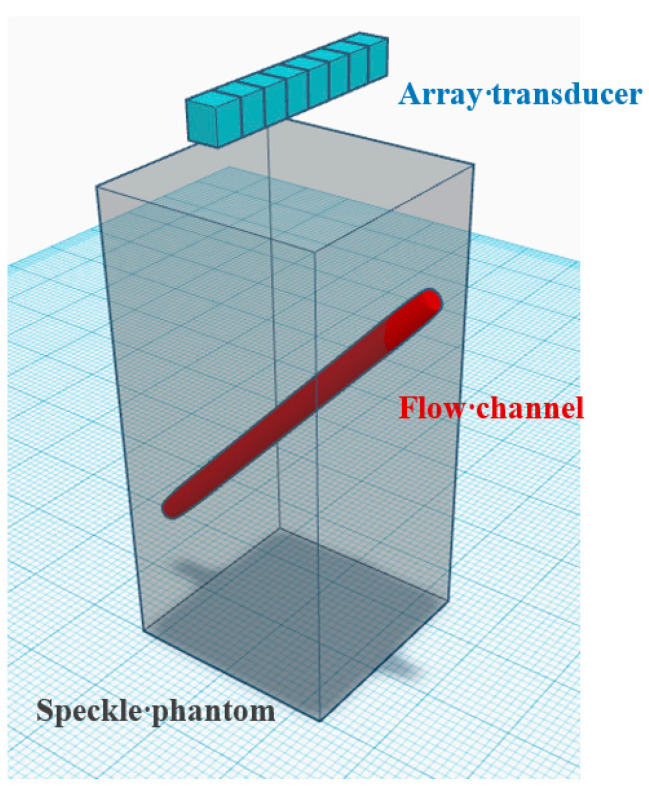
Schematic diagram of the simulated flow channel in the speckle phantom. Note that the intersection of the image plane of the array transducer and the cylindrical vessel will be an ellipse.

The Field II program [30,31] has been used for all simulations. The simulation schematic is shown in Figure 2. A flow channel with a radius of 2 mm is embedded in the speckle-generating tissue phantom to simulate the blood vessel with an inclined angle of 45°. The scatterers inside the flow channel are assumed to move according to a parabolic velocity distribution (i.e., laminar flow) with the peak velocity at the center of 15 mm/s. The scatterer density is set to contain about 10 scatterers per resolution cell and the scattering magnitude of the tissue is assumed to be 60 dB higher than that of the blood flow in the flow channel. White Gaussian noises are included into the simulated channel waveforms before beamforming to achieve a channel signal-to-noise ratio of 0 dB for the blood flow signal. A 128-elements linear array transducer was used for both transmission and reception in the simulations. The transmit frequency is set to be 5 MHz. A total of 7 plane waves evenly spanning an azimuthal angular range of −7.5° to +7.5° are sequentially transmitted with a pulse-repetition-frequency (PRF) of 3.9 kHz to produce low-resolution images of size 375 × 128 from distinct PW angles. Therefore, the compounded high-resolution imaging has a frame rate of approximately 556 Hz. Note that the low-resolution images are constructed using baseband DAS beamforming in the direction of receiving channels as conventional CPWC imaging. The PW transmit sequence is repeated 15 times to provide an ensemble number of 15. Other detailed parameters are shown in Table 1. For each PW transmit angle, the corresponding low-resolution images from different ensembles are clustered in the ensemble direction to form a three-dimensional matrix of 375 × 128 × 15 for SVD clutter filtering to eliminate high-frequency noise and stationary tissue. In the simulation, the low-order and high-order thresholds of SVD clutter filter are set to 2 and 10, respectively. Finally, the filtered low-resolution images of different PW transmit angles were compounded using either DAS or DMAS processing to produce the high-resolution power Doppler image with ensemble averaging.

### 3.2. In Vivo Experimental Setup

**Figure 3:**
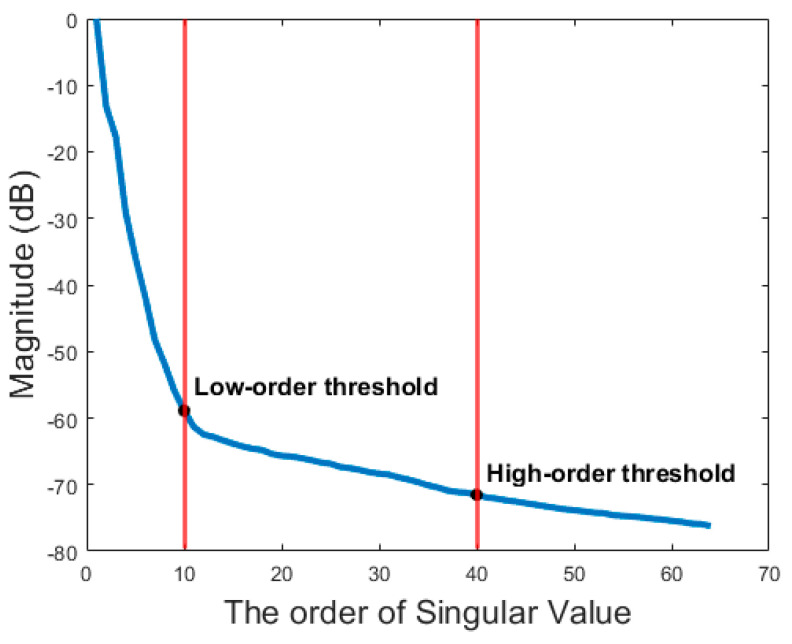
The curve of singular value of the in vivo experimental data and the corresponding low-order and high-order threshold for SVD clutter filtering.

The animal data was provided by the S-Sharp Corporation (New Taipei, Taiwan). The data was collected from a 6-month-old female New Zealand white rabbit. The rabbit was anesthetized with an intramuscular injection of Zoletil^®^ 50 according to its body weight and placed on a warming pad to maintain its body temperature at 37 °C. The L154BH linear array with a center frequency of 6.4 MHz was used in the experiment. The experimental PW transmit sequence comprises 6 PW angles which equidistantly increasing from −5° to +5° with a PRF of 4 kHz. The corresponding low-resolution images were beamformed by Prodigy ultrasonic imaging system (S-sharp, New Taipei City, Taiwan) and then processed offline using Matlab (The MathWorks, Natick, MA, USA) for SVD clutter filtering. Therefore, the effective high-resolution frame rate is about 667 Hz. A total of 64 ensembles were acquired and each ensemble is compounded from low-resolution images from 6 angles. The detailed imaging parameters are shown in Table 2. In the in vivo experiment, the low-order and high-order thresholds of SVD clutter filter are set to be 10 and 40, respectively. These thresholds are determined using the descendent magnitude of each singular value. One typical example in Figure 3 demonstrates that the low-order threshold is regarded as the turning point at which the curve of singular value has a slope of −1 to indicate the beginning of the flattened curve. On the other hand, the high-order threshold corresponds to where the curve of singular value is about to decrease linearly. This is because the high-order singular value of white Gaussian noise should follow a linear distribution under the logarithm scale [32]. Similar to the simulations, the SVD clutter filter is also individually applied to the low-resolution images of each PW transmit angle. Then, these filtered low-resolution images are coherently compounded using either DAS or DMAS beamforming to produce the final high-resolution power Doppler image.

### 3.3. Quantitative Analysis

In order to quantitatively compare the image quality among different beamforming methods in power Doppler imaging, two region-of-interests (ROIs) are defined in the power Doppler image to respectively represent the blood flow area and the background area. The calculation of Doppler signal-to-noise ratio (SNR) and contrast-to-noise ratio (CNR) are defined as follows [33]:

$$SNR\;=\;10\cdot \log_{10}\left(\frac{{\bar{M}}_{blood}}{{\bar{M}}_{background}}\right)\;CNR\;=\;10\cdot \log_{10}\left(\frac{\left|{\bar{M}}_{blood}\;-\;{\bar{M}}_{background}\right|}{\sigma_{background}}\right)$$

where ${\bar{M}}_{blood}$ and ${\bar{M}}_{background}$ are the mean power of blood flow and background signals, respectively, and $\sigma_{background}$ represent the standard deviation of background signals. For each power Doppler image in this study, its leftmost upper panel shows the corresponding ROIs for the blood flow region (blue box) and the background region (white box).

## 4. Results

### 4.1. Simulations

**Figure 4:**
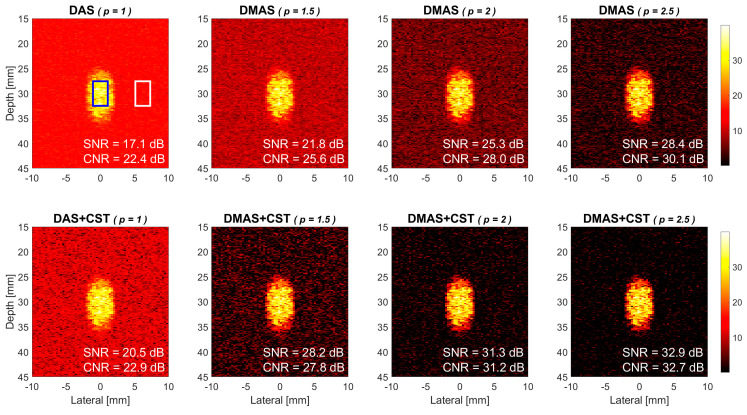
Simulated power Doppler images of flow phantom for DAS beamforming, DMAS beamforming with the *p* value of 1.5, 2.0 and 2.5, respectively from left to right. Seven PW transmit angles are used (i.e., [−7.5° −5° −2.5° 0° +2.5° +5° +7.5°]). Upper panels: without the CST technique. Lower panels: with the CST technique.

Power Doppler images of the simulated flow phantom in DMAS beamforming and DMAS-CST beamforming are respectively provided in the upper and lower panels of Figure 4. Seven PW transmit angles are used for coherent compounding (i.e., [−7.5° −5° −2.5° 0° +2.5° +5° +7.5°]). The flow velocity in the simulation is 15 mm/s and the ensemble number for averaging is 15. The power Doppler images from left to right correspond to different *p* values of 1.5, 2.0 and 2.5 in both DMAS and DMAS-CST beamforming while DAS beamforming is also provided as a reference in the leftmost panels. Note that the power level of Doppler image is represented using the brightness as shown in the color bar. A brighter image pixel means that the Doppler power in this spatial location is higher than that in other pixels. The background region (i.e., the white box) does not enclose any flow vessel and thus its power only comes from the random noises. Since the background region of DMAS image appears to be darker than that of DAS image, visual observations indicate that the power Doppler images with DMAS beamforming alone generally have a lower noise level in the background than that with DAS beamforming. Specifically, the Doppler SNR increases from 17.1 dB in DAS to 21.8 dB, 25.3 dB, and 28.4 dB in DMAS, respectively, with the *p* value of 1.5, 2.0, and 2.5. Take the *p* value of 2.0 in DMAS beamforming as an example, the improvement in Doppler SNR is 8.2 dB compared to the DAS counterpart. On the other hand, when DMAS beamforming is performed together with the CST technique, it is also apparent in Figure 4 that the background noise can be further suppressed to a lower level in DMAS-CST beamforming. The corresponding Doppler SNR improves by another 6.4 dB, 6.0 dB, and 4.5 dB, respectively, for the *p* value of 1.5, 2.0, and 2.5. Note that the improvement in Doppler SNR due to the CST technique appears to decrease with the *p* value in DMAS beamforming. This observation will be discussed later. Note that DAS beamforming with CST technique can also improve the Doppler SNR but only by 3.4 dB.

**Figure 5:**
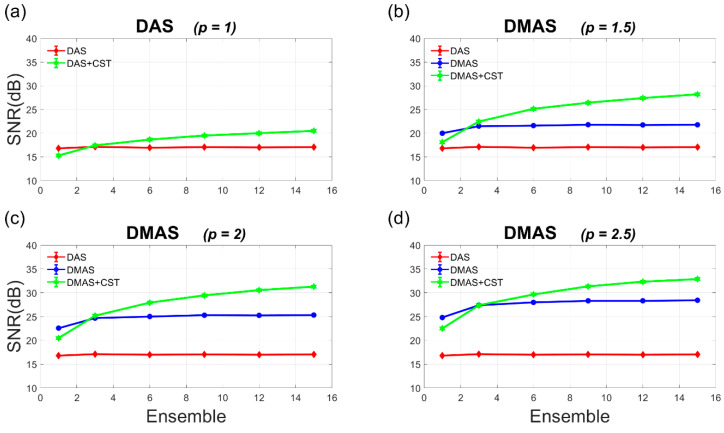
Quantitative analysis of Doppler SNR in the simulations as a function of ensemble number for (**a**) DAS beamforming and (**b**–**d**) DMAS beamforming with the *p* value of 1.5, 2.0, and 2.5, respectively.

**Figure 6:**
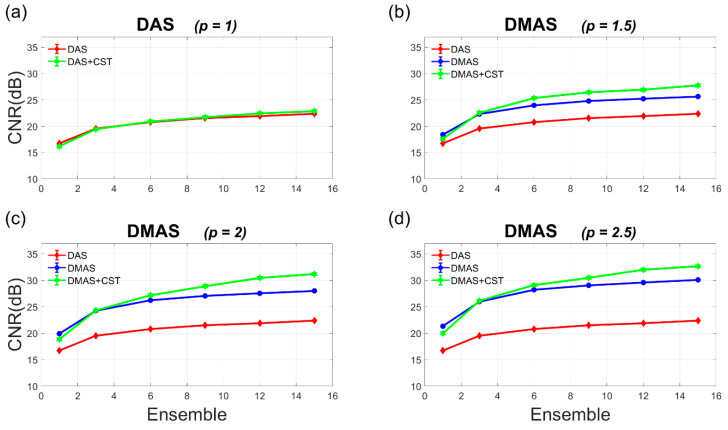
Quantitative analysis of Doppler CNR in the simulations as a function of ensemble number for (**a**) DAS beamforming and (**b**–**d**) DMAS beamforming with the *p* value of 1.5, 2.0, and 2.5, respectively.

The Doppler SNR and CNR are also provided as a function of ensemble number ranging from 1 to 15 in Figure 5 and Figure 6. In Figure 5, it should be noted that the Doppler SNR without the CST technique generally remains unchanged with the ensemble number in both DAS and DMAS beamforming. This is because the incoherent summation of power Doppler ensembles only helps to smooth the noise variation in the background but is not able to suppress the noise level. With the CST technique, on the contrary, the achievable Doppler SNR in both DAS-CST and DMAS-CST beamforming appears to consistently increase with the ensemble number. This is as expected since the CST technique depends on a diagonal autocorrelation matrix among PW transmit angles (i.e., **R** in Equation (6)) to remove the uncorrelated random noises. Nonetheless, for random noises, it takes sufficient realizations (i.e., sufficient power Doppler ensembles) for the autocorrelation matrix to converge to the diagonal form. This is why the SNR improvement due to the CST technique would increase with the ensemble number. Take DMAS beamforming with *p* value of 2.0 as an example, the CST technique improves the Doppler SNR by 2.9 dB and 6.0 dB, respectively, when the ensemble number is 6 and 15. Similarly, the Doppler SNR in DAS beamforming also improves by 1.7 dB and 3.4 dB due to the CST technique, respectively for the ensemble number of 6 and 15. On the contrary, the Doppler CNR appears to increase with the ensemble number no matter whether the CST technique is performed or not. This comes from the reduction of noise variation in the process of ensemble averaging. For DAS beamforming, however, the Doppler CNR without and with the CST technique almost overlap with each other. In other words, the CST technique barely improves the Doppler CNR in DAS beamforming. For DMAS beamforming, on the other hand, the Doppler CNR markedly increases due to the CST technique for all *p* values considered here. Take the *p* value of 2.0 as an example, the CST technique improves the Doppler CNR in DMAS beamforming by 0.9 dB and 3.2 dB, respectively when the ensemble number is 6 and 15. Nonetheless, it should be noted that the CST technique could adversely lead to the decrease of Doppler SNR and CNR when the ensemble number is small (e.g., smaller than three in the simulation as shown in Figure 5 and Figure 6).

**Figure 7:**
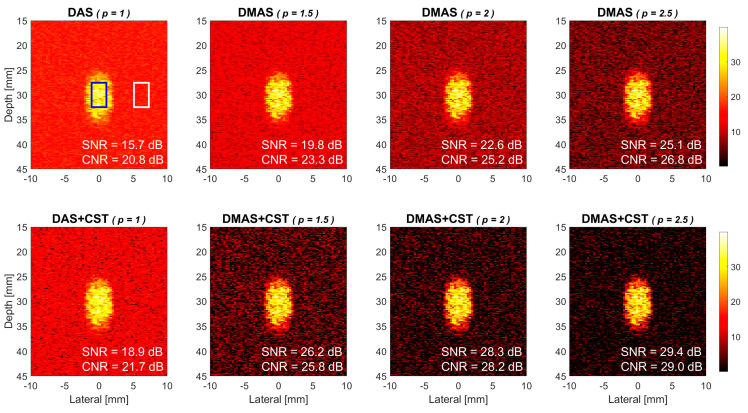
Simulated power Doppler images of flow phantom for DAS beamforming, DMAS beamforming with the *p* value of 1.5, 2.0 and 2.5, respectively from left to right. Five PW transmit angles are used (i.e., [−5° −2.5° 0° +2.5° +5°]). Upper panels: without the CST technique. Lower panels: with the CST technique.

Since the DMAS-based power Doppler detection in this study relies on the signal coherence among low-resolution images from different PW transmit angles, the effect of the number of PW transmit angle should be considered. Figure 7 shows that the simulated power Doppler images when the number of PW transmit angle is reduced to 5 (i.e., [−5° −2.5° 0° +2.5° +5°]). All other imaging parameters remain the same as those in Figure 4. Compared to its 7-angle counterpart in Figure 4, it should be noted that the 5-angle power Doppler images in Figure 7 exhibit higher background noise level and the resultant Doppler SNR and CNR also decrease relative to those in Figure 4. For example, the Doppler SNR in DMAS beamforming without CST technique decreases from 21.8 dB, 25.3 dB, and 28.4 dB in Figure 4 to 19.8 dB, 22.6 dB, and 25.1 dB in Figure 7, respectively, with the *p* value of 1.5, 2.0, and 2.5. In other words, when a smaller number of PW transmit angle is used to construct the final high-resolution power Doppler image for a higher frame rate, the Doppler SNR and CNR may decrease in DMAS beamforming. This implies that the coherence-based suppression of random noises performs better when more realizations of noise are available from distinct PW transmit angles. In DMAS-CST beamforming, on the other hand, the Doppler SNR also decreases from 28.2 dB, 31.3 dB, and 32.9 dB in Figure 4 to 26.2 dB, 28.3 dB, and 29.4 dB in Figure 7. Note that the Doppler SNR in DAS beamforming also decreases from 17.1 in Figure 4 to 15.7 in Figure 7. However, it should be taken into considerations that the ensemble number of power Doppler image is fixed to 15 for both 5-angle and 7-angle transmit sequences in this comparison. In practical applications, DMAS-CST beamforming may be expected to suffer less from a smaller number of PW transmit angle and will be discussed later.

**Figure 8:**
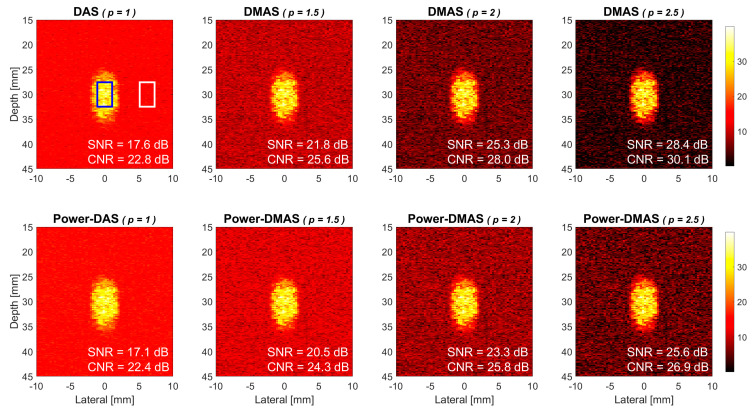
Simulated power Doppler images of flow phantom for DAS beamforming, DMAS beamforming with the *p* value of 1.5, 2.0, and 2.5, respectively, from left to right. Seven PW transmit angles are used (i.e., [−7.5° −5° −2.5° 0° +2.5° +5° +7.5°]). Upper panels: ensemble averaging of signal magnitude. Lower panels: ensemble averaging of signal power.

It has been clearly indicated in the theory section that the DMAS-based power Doppler in this study is performed by averaging the magnitude of Doppler signal among different ensembles and then the power of the averaged Doppler signal is estimated as in Equation (5). This is different from the conventional approach in which the power of Doppler signal is averaged among ensembles as in Equation (1). The reason for performing ensemble averaging of signal magnitude instead of signal power can be justified by the power Doppler images as shown in Figure 8. In the upper panels, the power Doppler images are constructed using ensemble averaging of signal magnitude for both DAS beamforming and DMAS beamforming with the *p* value of 1.5, 2.0, and 2.5, respectively, from left to right. In the lower panels, however, the power Doppler images are constructed using ensemble averaging of signal power as in the conventional approach. Therefore, some of the panels in Figure 8 are just duplicates of those in Figure 4. It is apparent that the power Doppler images in the upper panels consistently have a lower noise level than their counterpart in the lower panels, especially for DMAS beamforming. Specifically, the Doppler SNR in DMAS beamforming improves by 1.3 dB, 2.0 dB, and 2.8 dB, respectively for *p* values of 1.5, 2.0, and 2.5 when the ensemble averaging is switched from power to magnitude. Note that the Doppler SNR in DAS beamforming also improves by 0.5 dB but this minor change in noise level is not visually detectable in the corresponding power Doppler images.

### 4.2. Experiments

**Figure 9:**
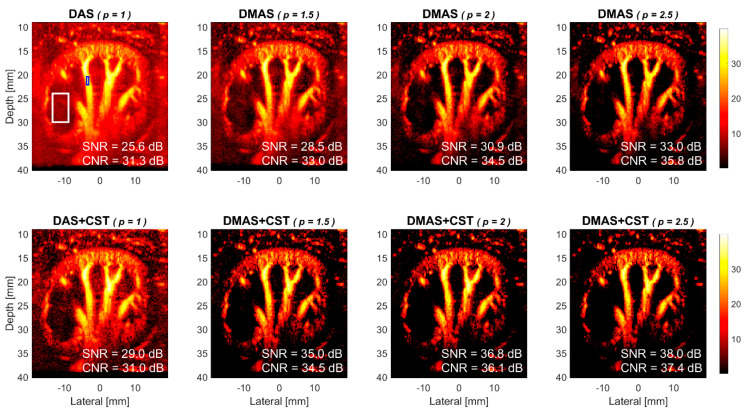
Experimental power Doppler images of rabbit’s kidney for DAS beamforming, DMAS beamforming with the *p* value of 1.5, 2.0, and 2.5, respectively from left to right. Six PW transmit angles are used (i.e., [−5° −3° −1° +1° +3° +5°]). Upper panels: without the CST technique. Lower panels: with the CST technique.

Experimentally acquired power Doppler images of the rabbit’s kidney are provided in Figure 9 for DAS and DMAS beamforming without and with CST technique. Visual observations also demonstrate that the power Doppler images with DMAS beamforming generally has a lower noise level in the background than that with DAS beamforming. These observations on experimental images are in agreement with those on simulations. Specifically, the experimental Doppler SNR without CST technique increases from 25.6 dB in DAS to 28.5 dB, 30.9 dB, and 33.0 dB in DMAS, respectively, with the *p* value of 1.5, 2.0, and 2.5. Take the *p* value of 2.0 in DMAS beamforming as an example, the improvement in experimental Doppler SNR is 5.3 dB compared to the DAS counterpart. On the other hand, when DMAS beamforming is performed together with CST technique, it is also apparent in Figure 9 that the background noise can be further suppressed to a lower level in DMAS-CST beamforming. Specifically, the experimental Doppler SNR in DMAS-CST beamforming improves by 6.5 dB, 5.9 dB, and 5.0 dB, respectively, with the *p* value of 1.5, 2.0, and 2.5, compared to those in DMAS beamforming alone. The efficacy of CTS technique on alleviating uncorrelated noises is also consistent between the experimental and the simulation results. Besides, though the CST technique in the experiments does help to further boost the Doppler SNR, the achievable improvement also decreases with the *p* value in DMAS beamforming.

**Figure 10:**
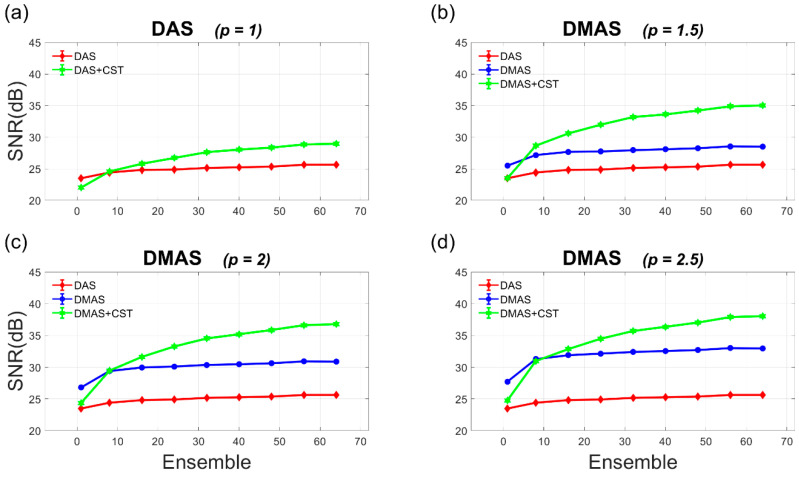
Quantitative analysis of Doppler SNR in the experiments as a function of ensemble number for (**a**) DAS beamforming and (**b**–**d**) DMAS beamforming with the *p* value of 1.5, 2.0, and 2.5, respectively.

**Figure 11:**
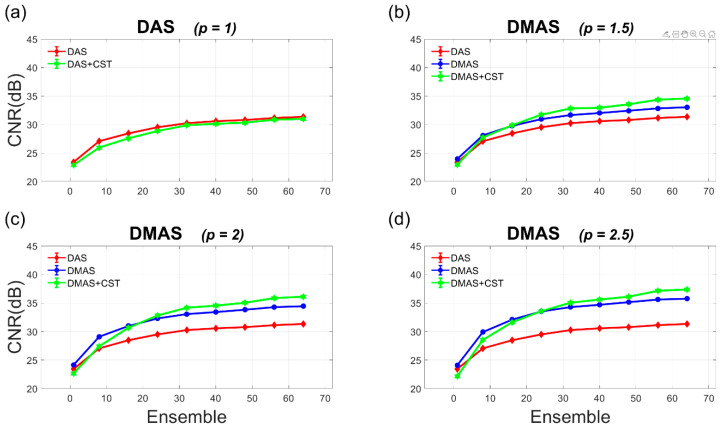
Quantitative analysis of Doppler CNR in the experiments as a function of ensemble number for (**a**) DAS beamforming and (**b**–**d**) DMAS beamforming with the *p* value of 1.5, 2.0, and 2.5, respectively.

The experimental Doppler SNR and CNR are quantitatively provided as a function of ensemble number ranging from 1 to 64 in Figure 10 and Figure 11. It should be noted that the experimental Doppler SNR without the CST technique in Figure 10 generally remains unchanged with the ensemble number for both DAS and DMAS beamforming. This phenomenon agrees with the simulation results in Figure 5. In contrast, when the CST technique is performed together with either DAS or DMAS beamforming, the experimental Doppler SNR increases with the ensemble number. This is also consistent with that in the simulations because sufficient power Doppler ensembles would allow the autocorrelation matrix to be diagonal for the CST technique to remove uncorrelated noises. With the *p* value of 2.0, the experimental Doppler SNR improves from 30 dB, 30.3 dB, and 30.9 dB in DMAS beamforming alone to 31.6 dB, 34.5 dB, and 36.8 dB in DMAS-CST beamforming, respectively, when the ensemble number increases from 16, 32, and 64. When the ensemble number is small, however, it should be noted that the CST technique may adversely compromise both Doppler SNR and Doppler CNR. For example, with the *p* value of 2.0, the Doppler SNR with only one ensemble actually decreases from 26.8 dB in DMAS beamforming to 24.3 dB in DMAS-CST beamforming. This observation also agrees with that in simulations. Actually, Figure 10 shows that the CST technique demands an ensemble number larger than eight in the experiments to provided improvement in Doppler SNR for both DAS and DMAS beamforming.

In contrast, the Doppler CNR in Figure 11 increases with the ensemble number due to the reduced variation of noise no matter whether the CST technique is performed or not for both DAS and DMAS beamforming. For DAS beamforming, however, the CST technique appears to barely improve the Doppler CNR for all number of ensembles. For DMAS beamforming, on the other hand, the CST technique with sufficient ensembles could provide noticeable improvement in Doppler CNR due to the suppressed noise background. For example, the CST technique improves the Doppler CNR in DMAS beamforming by 1.2 dB and 1.6 dB, respectively, with the ensemble number of 32 and 64 when the *p* value is 2.0.

## 5. Discussion and Conclusions

In this study, DMAS beamforming of low-resolution images from distinct PW transmit angles is used to construct a novel coherence-based power Doppler detection in multi-angle PW imaging. Moreover, the CST technique is also developed to further reduce the noise level in power Doppler detection by correlation of two DMAS signals from complementary subset transmit. Since the proposed method is based on signal coherence of the echo matrix in the dimension of PW transmit angle, it can be readily applied to boost the performance of conventional CPWC imaging by replacing DAS beamforming of low-resolution images with DMAS beamforming. Note that the proposed DMAS beamforming of low-resolution images does not require the raw channel data and thus is relatively free from huge memory allocation to record the entire echo matrix in multi-angle PW imaging. Specifically, DMAS beamforming in the dimension of PW transmit angle is performed by first maintaining the phase of low-resolution pixel but adopting the *p*-th root to scale the pixel magnitude. After the summation of magnitude-scaled low-resolution image pixels from available PW transmit angles, the *p*-th power is performed to produce the final high-resolution image pixel. Here, the *p* value represents the degree of signal coherence considered in DMAS beamforming and thus a higher *p* value generally produces higher-quality images. For the implementation of CST technique, complementary transmit subsets can be defined from the available PW transmit angles to produce the corresponding signals in DMAS beamforming. Then, the two DMAS signals can be correlated to reduce the noise level in the final power Doppler imaging. Note that the CST technique is also applicable to DAS beamforming by correlating two complementary DAS signals from conventional CPWC imaging.

**Figure 12:**
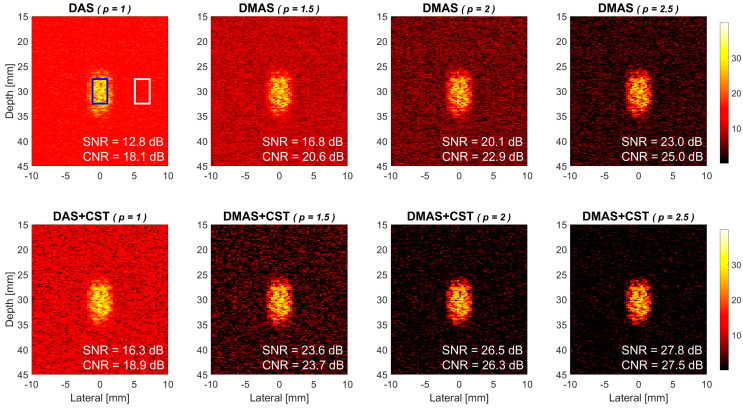
Simulated power Doppler images of flow phantom for DAS beamforming, DMAS beamforming with the *p* value of 1.5, 2.0, and 2.5, respectively from left to right. Upper panels: without the CST technique. Lower panels: with the CST technique. All simulation parameters and signal processing remain the same as those in Figure 4 except that the peak flow velocity is reduced from 15 mm/s to 5 mm/s to produce a weaker flow after clutter filtering. Note that the brightness of each panel is normalized to that of its counterpart in Figure 4.

It should be emphasized that both the DMAS beamforming and the CST technique improve the quality of power Doppler image by including the signal coherence into the image output. They are intrinsically different from a simple nonlinear mapping of the pixel value which would darken any low-intensity pixel regardless of whether the pixel belongs to noise or blood flow and thus degrade the image contrast. In order to validate this, a weaker flow is simulated as shown in Figure 12 by reducing the peak velocity to only 5 mm/s while all other simulation parameters and signal processing remain unchanged to those in Figure 4. Note that the weaker flow intensity is demonstrated both by the lower brightness of the flow region and the corresponding Doppler SNR in Figure 12 than its counterpart in Figure 4 for each panel. With the same noise level in the simulation of both Figure 4 and Figure 12, their difference in Doppler SNR actually represents the image contrast of power Doppler detection between the stronger and the weaker flow signals. Take the DMAS-CST beamforming in the lower panels as an example, the Doppler SNR decreases from 28.2 dB, 31.3 dB, and 32.9 dB in Figure 4 to 23.6 dB, 26.5 dB, and 27.8 dB in Figure 12, respectively, with the *p* value of 1.5, 2.0, and 2.5. Therefore, the image contrast of DMAS-CST beamforming between Figure 4 and Figure 12 is respectively 4.6 dB, 4.8 dB, and 5.1 dB. Compared to the DAS reference whose image contrast is 4.3 dB (i.e., 17.1–12.8), DMAS-CST beamforming exhibits no significant change in image contrast with marked suppression in background noises. In other words, the proposed DMAS-CST beamforming can preserve the image contrast of conventional DAS beamforming while improving the Doppler SNR significantly.

Moreover, in the proposed DMAS and DMAS-CST beamforming, the Doppler power is estimated using a square-of-sum approach as defined in Equations (5) and (7). Compared to the conventional sum-of-square power estimation in Equations (1) and (3), the square-of-sum approach additionally includes the cross-correlation among ensembles into the estimated Doppler power. Therefore, the square-of-sum power can be understood as the sum-of-square power compensated by the cross-correlation among ensembles. Note that, with a sufficient number of ensembles, the cross-correlation term will statistically approach zero for uncorrelated thermal noises but will remain large for true flow signal. This is exactly why the averaging of signal magnitude before taking power (i.e., square of sum) in upper panels of Figure 8 always provides higher Doppler SNR and CNR than the averaging of signal power (i.e., sum of square) in the corresponding lower panels. This observation is also similar to that reported in [34] which uses larger lag of autocorrelation to represent the Doppler power.

Both simulations and experiments have been performed to validate the DMAS-based power Doppler imaging. Results indicate that, since the random noises have a low coherence among low-resolution images, the proposed DMAS beamforming is capable of producing a lower background noise level than the DAS counterpart and thus the achievable Doppler SNR increases with the *p* value in DMAS beamforming. Besides, when the CST technique is integrated with DMAS beamforming, the corresponding Doppler SNR further improves by another 6.4 dB, 6.0 dB, and 4.5 dB in the simulations for DMAS-CST beamforming with the *p* value of 1.5, 2.0, and 2.5, respectively. Note that the improvement in Doppler SNR due to the CST technique decreases with the *p* value. Our experimental results also confirm the decrease of achievable improvement in Doppler SNR with the *p* value in DMAS-CST beamforming. This is because the DMAS beamforming without CST technique already helps to suppress not only the low-coherence image clutter but also the uncorrelated random noises. Consequently, when the random noises have been largely suppressed by adopting a higher *p* value in DMAS beamforming, there will be fewer residual noises left for the CST technique to remove. This is probably why the efficacy of CST technique on Doppler SNR appears to degrade with the increasing *p* value in DMAS-CST beamforming. On the other hand, the CST technique in DAS beamforming barely leads to any improvement in Doppler CNR, as demonstrated by the overlap of Doppler CNR without and with CST technique in both Figure 6a and Figure 11a. This observation is consistent with that reported in [28] even though their complementary subsets are defined in the receiving aperture while ours are defined in the PW transmit angle. Nonetheless, it should be noted that the CST technique in DMAS beamforming does provide a marked improvement in Doppler CNR. Moreover, in order to remove the random noises effectively, the CST technique demands sufficient ensembles to ensure the diagonal autocorrelation matrix of noises from distinct PW transmit angles. Consequently, the CST technique may adversely degrade the quality of power Doppler detection when the number of ensembles is small. As the two complementary weighting vectors $w_{1}$ and $w_{2}$ are selected to respectively correspond to the negative and the positive PW transmit angles in this study, it can be generalized to any complementary pair. Theoretically, the complementary pair with interleaved PW transmit angles should be preferred to minimize the angle difference between the two subsets. This is because, when the imaged features have a certain orientation, a large difference in PW transmit angle between the two subsets could make the imaged features more visible in one transmit subset than the other. In this case, these particular features will be relatively suppressed by the correlation of the two complementary DMAS signals as compared to other features without obvious orientations.

Our results also indicate that the performance of DMAS-based power Doppler imaging would improve with the number of PW transmit angles. This is because the image clutter and noises can be better distinguished from the true flow signal by comparing among the low-resolution image pixels from more PW transmit angles. Nonetheless, it should be noted that the aforementioned observation is based on the same number of ensemble for two PW transmit sequences with different number of PW transmit angle. In practical applications, the number of PW transmit angle is actually related to the achievable number of ensemble for averaging. For example, with a temporal window of 1 s for Doppler detection and a PRI of 100 μs, the number of high-resolution ensembles will be 2000 and 2500, respectively for a PW transmit sequence with 5 angles and 4 angles. For DMAS beamforming without CST technique, since the corresponding Doppler SNR in both simulations and experiments generally remains unchanged with the number of ensemble, the 5-angle PW transmit sequence will be preferred due to its larger number of PW angles for better coherence estimation in DMAS beamforming. For DMAS-CST beamforming, on the contrary, the corresponding Doppler SNR noticeably increase with the number of ensemble and thus the advantage of the 5-angle PW transmit sequence could be compromised by its smaller number of ensemble compared to that of the 4-angle PW transmit sequence. In other words, DMAS-CST beamforming may suffer less from the smaller number of PW transmit angle due to the corresponding increase in the number of ensemble.

One major limitation of DMAS-based power Doppler imaging may be its computational efficiency. Since the proposed DMAS beamforming involves multiplicative operation of the low-resolution images from distinct PW transmit angles, the low-resolution images have to be firstly grouped according to its PW transmit angle and then each group is individually band-pass filtered in the direction of ensemble using SVD to remove the stationary tissue before DMAS beamforming. Otherwise, if the band-pass filtering is performed after DMAS beamforming, the multiplicative coupling between the blood flow and stationary tissue will be no longer removable. Consequently, the band-pass filtering has to be repetitively performed by *M* times where *M* is the total number of PW transmit angle for DMAS beamforming. For DAS beamforming (i.e., CPWC imaging), on the other hand, its linear operation allows the band-pass clutter filter to be implemented in the final high-resolution images to ease the computational burden. Note that, however, the computational complexity in clutter filtering increases not only for the proposed DMAS beamforming but also for any nonlinear beamforming such as CFPD in [21]. In this case, a simpler filter such as Finite Impulse Response may be preferred instead of the SVD filter in this study for real-time implementation of DMAS-based power Doppler imaging.

## Figures and Tables

**Table 1:** The imaging parameter of Field II simulations.

**Imaging System**	
Transducer	Linear Array
Pitch	0.3 mm
Number of elements	128
Elevation focus	30 mm
Sampling frequency	20 MHz
Image size in pixels	375 (axial) × 128 (lateral)
**Transmit Pulse**	
Center frequency	5.0 MHz
Excitation	3 cycles
PW transmit angle	7 (−7.5°~+7.5°)
Ensemble	15
PRF	3.9 kHz
**Phantom**	
Speed of Sound	1540 m/s
Scattering magnitude	60 dB (tissue clutter)
	0 dB (blood flow)

**Table 2:** The imaging parameter of in vivo experiment.

**Prodigy Imaging System**	
Transducer	L154BH
Pitch	0.3 mm
Number of elements	128
Elevation focus	20 mm
Sampling frequency	25.6 MHz
Image size in pixels	520 (axial) × 128 (lateral)
**Transmit Pulse**	
Center frequency	6.4 MHz
Excitation	5 cycles
PW transmit angle	6 (−5°~+5°)
Ensemble	64
PRF	4 kHz

## Data Availability

Restrictions apply to the availability of these data. Experimental data was obtained from S-Sharp Corporation (New Taipei, Taiwan) and are available with the permission of S-Sharp Corporation.
